# The development of a high-throughput acoustic droplet ejection mass-spectrometry assay and a solid-supported membrane (SSM)-based electrophysiological assay to study the pharmacological inhibition of SLC1-A3, -A2 and -A1 in a drug discovery program

**DOI:** 10.3389/fphar.2025.1544682

**Published:** 2025-04-16

**Authors:** Yasmin Zuschlag, Antje Pommereau, Jennifer Warkentin, Thomas Licher, Felix Bärenz

**Affiliations:** Sanofi, Integrated Drug Discovery, Frankfurt, Germany

**Keywords:** drug discovery, excitatory amino acid transporters (EAATs), high content screening, acoustic droplet ejection mass-spectrometry, SSM-based electrophysiology

## Abstract

**Introduction:**

The solute carrier (SLC) family comprises a diverse group of membrane proteins essential for transporting a variety of substrates across cellular membranes. These transporters play crucial roles in cellular homeostasis, nutrient uptake, and neurotransmitter clearance. The SLC1 subfamily, specifically SLC1A3 (EAAT1), SLC1A2 (EAAT2), and SLC1A1 (EAAT3), are excitatory amino acid transporters that regulate glutamate concentrations in the synaptic cleft, making them important targets for neurological disorder therapeutics. Despite their significance, drug discovery efforts targeting these transporters have been hampered by limitations in available screening methodologies.

**Methods:**

We are utilizing advanced methodologies such as Acoustic Droplet Ejection Mass Spectrometry (ADE‐MS) and Solid Supported Membrane (SSM)-based electrophysiology to develop assays for the SLC1 family members: SLC1A3 (EAAT1), SLC1A2 (EAAT2), and SLC1A1 (EAAT3).

**Results and Discussion:**

In this manuscript, we present the successful development of novel assays specifically designed for drug discovery applications targeting the SLC1 family members. Our Acoustic Droplet Ejection Mass Spectrometry (ADE‐MS) platform demonstrated high sensitivity and reproducibility in detecting substrate transport activity across all three transporters. The complementary Solid Supported Membrane (SSM)‐based electrophysiology assay provided real-time kinetic measurements of transporter function with minimal background interference. These assays exhibited Z’ factors exceeding 0.7, indicating their robustness for high-throughput screening campaigns. Initial validation using known inhibitors confirmed the assays’ ability to identify compounds with varying potencies and mechanisms of action against SLC1A3, SLC1A2, and SLC1A1.

**Conclusion:**

We endeavor to establish robust assays that can facilitate future drug discovery campaigns.

## Introduction

Maintaining cellular homeostasis and integrity, essential for overall cell survival, depends on the precise regulation of molecular exchange between intracellular and extracellular environments (Digles et al., 2024; Dvorak et al., 2021). Solute Carriers (SLCs) are the largest transporter superfamily in human cells, consisting of more than 455 members arranged into 66 families (Digles et al., 2024). They are responsible for the uptake and efflux of nutrients, ions, metabolites, and xenobiotics, and are vital for maintaining metabolic functions (Digles et al., 2024; Hediger et al., 2013; Ferrada and Superti-Furga, 2022). Hence, the SLC family plays a crucial role in many physiological processes.

Dysfunction in these transporters is associated with various diseases, including diabetes, gout, hypertension, asthma, inflammatory bowel disease, chronic kidney disease, cancer, inborn errors of metabolism, and mental and neurological disorders (Dvorak et al., 2021; Wang et al., 2020). This renders them significant subjects for the exploration of new drugs. However, compounds in clinical trials currently target only about 10 SLC classes, thereby indicating an enormous scope for drug development in forthcoming treatments (Wang et al., 2020).

The Solute Carrier Family 1 (SLC1), known as Excitatory Amino Acid Transporters (EAATs), are crucial for high-affinity, Na^+^-dependent glutamate uptake, essential for neurotransmission, learning, memory, and neuronal health (Freidman et al., 2020; Todd and Hardingham, 2020). Proper regulation of glutamate levels prevents excitotoxicity, a condition linked to neurodegenerative diseases (Freidman et al., 2020; Todd and Hardingham, 2020). There are five members of EAATs, with EAAT1 (SLC1A3) and EAAT2 (SLC1A2) accounting for 90% of glutamate uptake in the human central nervous system (Freidman et al., 2020). EAATs maintain low extracellular glutamate levels to prevent excitotoxicity and allow glutamate to serve as a signaling molecule in the brain (Magi et al., 2019).

Dysfunction or abnormal expression of glutamate transporters has been associated with various neurological and psychological disorders such as Alzheimer’s disease, Parkinson’s disease, epilepsy, schizophrenia, major depressive disorder (MDD), Huntington’s disease, multiple sclerosis, as well as cancer and chronic pain (Freidman et al., 2020; Gegelashvili and Bjerrum, 2019; Pajarillo et al., 2019; Parkin et al., 2018; Peterson and Binder, 2020; Temmermand et al., 2022; Todd and Hardingham, 2020). Mutations in the SLC1A3 gene can cause a particular type of episodic ataxia (EA), characterized by paroxysmal cerebellar incoordination combined with epilepsy and migraine-like headaches, referred to as episodic ataxia type 6 (Chivukula et al., 2020; Choi et al., 2017; de Vries et al., 2009; Iwama et al., 2018). Furthermore, severe forms of epileptic encephalopathy (EE), characterized by early onset and multiple seizure types combined with developmental slowing or even regression, are linked to mutations in the gene coding for EAAT2 (SLC1A2) (Kovermann et al., 2022). Some loss-of-function mutations have also been found in the SLC1A1 gene, causing issues with EAAT3 expression and resulting in dicarboxylic aminoaciduria (Bailey et al., 2011; Bianchi et al., 2014). Aging-related decline in glutamate uptake capacity underscores the importance of these transporters in neurodegenerative diseases (Todd and Hardingham, 2020).

Despite their importance in the medical field and their potential for therapeutic treatment, the majority of SLCs remain understudied (Dvorak et al., 2021). One of the key challenges is the scarcity of suitable tool compounds that can effectively support the development of robust, specific, and functional SLC assays for pharmaceutical drug discovery campaigns. The absence of tool compounds frequently poses challenges in validating functional assays, thereby hindering progress in developing drug-like compounds. It is imperative to address this scarcity to surmount these hurdles and propel advancements in the development of effective solutions within this domain (Digles et al., 2024).

In the context of SLC1 transporters, only a few compounds have proven effective in modulating glutamate transporters. Among these, derivatives of threo-ß-hydroxyaspartic acid (THA) have shown promise in modulating glutamate transporters. Compounds like threo-ß-benzyloxyaspartic acid (TBOA) and (L-threo)-3-[3-[4-(trifluoromethyl)benzoylamino]-benzyloxy]-aspartate (TFB-TBOA) have potential as potent inhibitors (Fu et al., 2018; Kato et al., 2022; Shimamoto et al., 2004). Inspired by TFB-TBOA, selective inhibitors like WAY-213613 and allosteric modulators such as UCPH-101 and UCPH-102 have been developed, showing potential for the development of effective assays for large-scale drug screening initiatives and the discovery of innovative therapeutic interventions for various disorders (Canul-Tec et al., 2017; Dvorak and Superti-Furga, 2023; Hansen et al., 2016; Haym et al., 2016; Kato et al., 2022; Wang et al., 2020).

In the pursuit of advancing drug discovery, the challenges posed not only by the lack of well-annotated, specific, potent tool compounds but also by the need for robust and functional assay platforms are undeniable. These obstacles have prompted the development of a cell-based ^13^C_5_, ^15^N-labeled glutamic acid uptake assay using the Acoustic Ejection Mass Spectrometry (ADE-MS) (Sciex, Darmstadt) with a high-throughput screening (HTS) focus. Furthermore, to deepen our understanding of the transporters, a medium-throughput biophysical assay has been devised for comprehensive characterization, leveraging the SSM-based electrophysiology platform SURFE^2^R 96 SE (Nanion Technologies, Munich). Both assay platforms have been shown to fulfill (high) throughput and robustness requirements for small molecule screening campaigns in drug discovery (DiRico et al., 2020; Dueñas et al., 2023; Gerbeth-Kreul et al., 2021; Pommereau et al., 2023; Simon et al., 2021; Speckmeier et al., 2022; Winter et al., 2023; Zacharias et al., 2023). The innovative integration of ADE-MS and SSM-based electrophysiology represents a significant advancement in methodologies, collectively establishing a powerful platform for in-depth analysis of membrane proteins and the drug discovery process.

Acoustic Droplet Ejection Mass Spectrometry represents a novel advancement in mass spectrometry, leveraging acoustic energy for non-contact sample introduction into electrospray ionization mass spectrometers. In this innovative method, micro- or nanoliter samples are dispensed into specialized multi-well plates optimized for acoustic coupling (Liu, 2022; Simon et al., 2021). A piezoelectric transducer generates a precisely controlled acoustic pulse, creating a focused pressure wave that ejects minute droplets, typically ranging from picoliters to nanoliters, towards an open port interface connected to the ESI source. This rapid desolvation process transforms analytes into charged ions that are then detected based on their mass-to-charge ratios (Dvorak et al., 2021; Speckmeier et al., 2022).

The advantages of ADE-MS are substantial; it operates 10 to 100 times faster than traditional liquid chromatography-mass spectrometry (LC–MS) techniques, significantly reducing analysis times and minimizing sample consumption—an essential benefit when handling scarce or costly samples (Liu, 2022; Winter et al., 2023). Furthermore, the non-contact ejection mechanism reduces the risk of cross-contamination, a notable drawback of conventional methods like matrix-assisted laser desorption/ionization mass spectrometry (MALDI-MS) (Speckmeier et al., 2022). However, it is crucial to recognize that ADE-MS requires careful optimization of acoustic parameters and may be sensitive to sample viscosity and heterogeneity, potentially limiting its use in certain scenarios (Speckmeier et al., 2022).

On another front, SSM-based electrophysiology, utilizing the SURFE^2^R platform, provides a robust, label-free methodology for the functional analysis of membrane proteins. This technique utilizes gold electrodes coated with self-assembled monolayers and lipid bilayers to create stable artificial membranes (Bazzone et al., 2017; Nanion Technologies, 2023). Membrane proteins, particularly challenging targets like solute carrier transporters, are integrated into these environments, allowing for the capture of transient capacitive currents generated during conformational changes or ion transport, initiated by solution exchanges (Bazzone et al., 2013; Pommereau et al., 2023). Compared to traditional patch-clamp electrophysiology, SSM-based methods enhance automation and high-throughput screening, although they may be limited to electrogenic proteins and may not fully replicate native conditions (Bazzone et al., 2013).

Integrating ADE-MS with SSM-based electrophysiology creates a powerful dual-platform approach that combines the rapid, high-throughput chemical profiling of ADE-MS with the functional insights of the SSM-based technique. This synergy allows for a direct correlation between chemical composition and biological function, enhancing drug discovery and our understanding of membrane protein activity. While both methods have limitations, such as sensitivity issues and applicability constraints, their combined strengths—speed, reduced sample volumes, and minimized cross-contamination—make this integrated approach a valuable asset in pharmaceutical research (Simon et al., 2021; Winter et al., 2023; Speckmeier et al., 2022; Nanion Technologies, 2023).

## Methods and materials

### Cell-based ^13^C_5_, ^15^N-labeled glutamic acid uptake assay

A day before the experiment, HEK293-JumpIn-cells overexpressing the respective Solute Carrier Transporter (control/HEK293-WT, SLC1A3/EAAT1, SLC1A2/EAAT2 and SLC1A1/EAAT3 by CeMM, Vienna) are first induced with 1 μg/mL Doxycycline (#33429, Sigma-Aldrich) and then plated into wells of a Poly-D-Lysin coated 384 well plate (#PP-0200, Beckmann Coulter, Krefeld) using a cell number of 15,000 cells/well performed with the Multidrop Combi Reagent Dispenser (Thermo Fisher Scientific). Afterward, the cells are incubated at room temperature for 15 min and centrifuged at 78 *g* for 1 min. Finally, the plate is incubated overnight at 37°C and 7.5% CO_2_.

On the day of measurement, the medium in the assay plates is discarded per Blue^®^Washer (BlueCatBio, Neudrossenfeld), and 10 µL of the potassium buffer containing 140 mM KCl (#P9333, Sigmal-Aldrich), 2 mM MgCl_2_ (#M2670, Sigma Aldrich), 20 mM Hepes (#H3375, Sigma-Aldrich), pH 7.4 (KOH, #1.09107 by Sigma-Aldrich) is added with the CyBio^®^ FeliX pipetting robot (Analytik Jena, Jena). In the case of compounds analysis, the potassium buffer contains the compounds in different concentrations, and an incubation step of 15 min at room temperature occurs. Afterward, 20 µL per well of the sodium buffer containing labeled ^13^C_5_,^15^N-glutamic acid (#607851, Sigma-Aldrich), 210 mM NaCl (#71376, Sigma-Aldrich; final concentration 140 mM), 2 mM MgCl_2_, 20 mM Hepes, pH 7.4 (NaOH (#1.09137, Sigma-Aldrich)) is added per CyBio^®^ FeliX. The plates are then incubated for 1 h at 37°C, 7.5% CO_2_, and 95% humidity. In the next step, the wells are washed with 70 µL/well potassium buffer using the Multidrop Dispenser, and the supernatant is discarded via Blue^®^Washer. Afterward, the precipitation process is started by adding 90 µL/well of precipitation agent (60% MeOH (#1053221, Fisher Chemicals), 29% Milli-Q^®^ H_2_O, 11% Formic Acid (#A117-50, Fisher Chemicals) with the CyBio^®^ FeliX pipetting robot. The plates are then shaken for 30 min at 500 rpm. Afterward, a centrifuge step at 863 × g is performed for 20 min. Finally, 40 µL/well is transferred on Echo^®^ MS qualified 384 well plates via the CyBio^®^ FeliX pipetting robot and then measured with the ADE-MS.

### ADE-MS assay readout

This technique utilizes acoustic energy to precisely eject droplets containing the sample into a mass spectrometer for analysis, providing high sensitivity and specificity. The feasibility of this technology as a suitable assay platform was previously described (Dueñas et al., 2023; Liu, 2022; Simon et al., 2021; Speckmeier et al., 2022; Wen et al., 2021; Winter et al., 2023; Zhang et al., 2021).

ADE-MS measurements were performed on an ADE-MS device (Sciex, Darmstadt) operated with Sciex OS software (version 2.1.6.59781). As carrier solvent, a mixture of 60% Acetonitrile (#10799704, Fisher Chemicals), 39% Milli-Q^®^ H_2_O, and 1% formic acid (#A117-50, Fisher Chemicals) were utilized with a constant flow rate of 300 μL/min. For all samples, an ejection volume of 5 nL (2 droplets) and a delay time of 2 s/well were used. For the detection of ^13^C_5_, ^15^N labeled glutamic acid, the Sciex mass spectrometer Triple Quad 6500+ is operated in positive mode, monitoring the multiple reaction (MRM) transition 154.1 → 136.1 *m/z* for labeled glutamic acid that is already published by Zhu et al. (2020) for LC/MS. The following settings are used: vaporizer temperature 450°C, spray voltage 5,500 V, ion source gas 1 90 psi, ion source gas 270 psi, curtain gas 20 psi, CAD gas 9 psi, dwell 95 ms, DP 35 V, EP 10 V, CE 14 V, CXP 18 V.

Data from the assay readouts were processed with Genedata Screener (version 18.0.2-Standard) or Excel. GraphPad Prism (version 10.1.2) is utilized for the graphical representation of the analyzed data.

The results of the conducted ^13^C_5_, ^15^N - glutamic acid uptake experiments are displayed as the integral of the area under the curve (AUC). During the assay development, experiments are conducted using multiple determinations to obtain reliable data sets. Results are displayed as the median of respective integral values. The error bars indicate the mean deviation (MD) of multiple determinations. For screening experiments, duplicates are conducted, and the results are displayed as the mean value of integral values, while error bars represent the standard deviation (SD). The screening results show the inhibitory activity of the compounds as a percentage of control (PoC). DMSO 1% was used as a high control, and the highly potent unselective inhibitor TFB-TBOA as a tool compound in a concentration of 1 µM as a low control for normalization. Dose-response curves are displayed by plotting the percentage of inhibitory activity of respective compounds on the EAAT1 transporter against increasing compound concentrations. IC_50_ values and confidence intervals, with a confidence level of 95%, are obtained through Genedata Screener^®^ analysis.

### Solid supported membrane-based electrophysiology

The innovative SURFE^2^R technology developed by Nanion Technologies was employed to assess the transport activity of the SLC1A3 transporter (EAAT1) through electrophysiological techniques. This approach utilizes solid-supported membranes to replicate the natural environment of the transporters, enabling comprehensive analysis of their functionality. This technique provides real-time data on transporter activity, playing a pivotal role in comprehending the intricacies of substrate interaction. Furthermore, previous work has underscored its potential as a highly specific and robust assay platform (Bazzone et al., 2013; 2017; 2022; Pommereau et al., 2023; Schulz et al., 2008).

Before the measurement takes place, the sensors need to be prepared. A 96-well sensor is used, with each well coated in a gold layer as a reference electrode (#182001, Nanion Technologies). The wells are filled with 100 µL/well of thiol solution consisting of 143 mg/L Octoadecanethiol (#O1858, Sigma-Aldrich) in isopropanol (#10091304, Fisher Chemicals) and incubated for 3 h. The sensor plate is then washed with the Blue^®^Washer, and the thiol solution is removed by centrifugation. The wells are washed twice with 100 µL/well isopropanol and twice with 100 µL/well Milli-Q^®^ H_2_O, with centrifugation between each washing step. The sensor is left uncapped for 15 min at room temperature to dry the residual H_2_O. The CyBio^â^ FeliX pipetting robot is then used to add 2 μL/well of the lipid solution consisting of 7.5 g/L 1,2-diphytanoyl-sn-glyero-3-phosphocholine (#850356, Avanti^®^ Polar Lipids Birmingham) in n-Decane (#111871000, Fisher Chemicals) onto the thiol-coated sensor, followed by 100 μL/well of potassium buffer containing (140 mM KCl, 2 mM MgCl_2_, 30 mM Hepes, pH 7.4 (KOH)).

Meanwhile, membrane fragments prepared by the protocol of Pommereau et al. (2023) are diluted with potassium buffer to the required concentration of 8 mg/mL. Next, the membrane fragments are sonicated using the Ultrasonic Processor (Dr. Hielscher GmbH, Teltow: cycle time: 0.5, amplitude: 20%). Subsequently, 5 μL/well of the membrane fragment solution is added to each well, and the sensor plate is centrifuged at 1811 *g* for 30 min. After an incubation of 1 h at RT, the measurement can be started. In the following conducted experiments, the *Double Solution Exchange* program is used.

When analyzing compounds, respective compounds are prepared in different dilution plates for each buffer in a 10-step dilution series and a dilution factor of 1:2, starting with the highest concentration of 30 µM. The integrated CyBio^®^ FeliX pipetting robot will then transfer the well content in 96-well format from the dilution plates of each buffer used in the program on the assay plate. After the compounds are added, they are further incubated for 15 min, the buffers are exchanged with the activation buffer containing 10.2 µM glutamic acid and the appropriate compound in different concentrations. Glutamic acid-triggered currents are recorded in a single run.

The SurfControl and DataControl software packages from Nanion Technologies record and analyze the measured currents. Raw data is exported as an Excel file (.xlsx) and analyzed in Excel using XLfit. GraphPad Prism (version 10.1.2) is utilized for the graphical representation of the analyzed data.

### Cell toxicity assay

To evaluate the cytotoxic effects of the compounds under investigation, the Cytotoxicity Detection Kit (LDH, Cat. No. 11 644 793 001, Roche) is used as a non-radioactive alternative to conventional assays, such as [3H]-thymidine incorporation and [51Cr]-release assays. This kit is designed as a precise, rapid, and user-friendly colorimetric assay to quantify cell death by measuring lactate dehydrogenase (LDH) activity released from compromised cells into the supernatant.

To prepare the Cytotoxicity Detection Kit (LDH), Vial 1 (Catalyst) is reconstituted by adding 1 mL of double-distilled water to the lyophilized powder, allowing it to dissolve for 10 min before thoroughly mixing. Vial 2 (Dye Solution) is brought to room temperature before use. The reaction mixture is then prepared based on the number of assays required: for 100 assays, 250 μL of reconstituted catalyst (Vial 1) is mixed with 11.25 mL of dye solution (Vial 2) and agitated thoroughly.

For this test, Hep G2 cells are utilized. The cells are maintained in continuous culture at 37°C, 7.5% CO_2_, and 95% humidity. Cells are cultured in DMEM (#41965-039, Gibco), supplemented with 25 mM HEPES (#15630-056, Gibco), 10% FBS (#AC-SM-0033, Anprotec), and 0.1% Gentamycin (#15750, Gibco). For long-term storage, Hep G2 cells are harvested after scale-up, resuspended in a cryoprotective solution of 90% FBS and 10% DMSO, and frozen at −80°C at a density of approximately 2 × 10^7^ cells/mL, with permanent storage in liquid nitrogen at −196°C.

For cell plating, a frozen vial containing 1.8 mL of Hep G2 cells (∼1 × 10^8^ cells/mL) is thawed at 37°C for 2 min. Cells are transferred to 50 mL of culture medium and centrifuged at 750 *g* for 4 min. After discarding the supernatant, the cell pellet is resuspended in fresh medium and plated in a 384-well AssayReadyPlate (#781090, Greiner) at a density of 15,000 cells per well in 50 µL. The plates are incubated without lids for 20–24 h at 37°C with 7.5% CO_2_ and 95% humidity.

For the LDH cytotoxicity assay, the plate containing pre-dispensed compounds (10 mM, 150 nL/well, resulting in a final concentration of 30 µM) is centrifuged at 1,100 rpm for 1 min. From each well, 20 µL of supernatant is transferred to a new assay plate with VPrep, followed by the addition of 20 µL of freshly prepared LDH reaction mixture with the Multidrop Combi (standard cassette). After incubation for 15–20 min at room temperature under a black lid, absorbance is measured at 492 nm using a PheraStar FSX plate reader.

### Matrix effect assay

To evaluate the influence of cell, substrate, or compound matrix effects on compound screening results, experiments were conducted using two different methods: one with EAAT1 cell lysates and one without.

In the cell lysate approach, 15,000 cells per well were seeded and incubated for 24 h in an incubator. The medium was subsequently removed using the Blue®Washer. Next, was a washing step with 70 µL/well of potassium buffer using the Multidrop Dispenser, followed by another buffer removal with the Blue®Washer. For cell lysis, 90 µL/well of a precipitation agent (60% MeOH, 29% H_2_O, 11% formic acid) was used. Increasing concentrations of ^13^C_5_ and ^15^N-glutamic acid (ranging from 0.15 to 300 µM) were added, along with 10 µM of the respective test compound. After preparing the samples, they were transferred to Echo-MS plates for measurement.

In the control approach without cell lysates, test compounds, and substrates were added directly to the precipitation agent and analyzed without prior cell culture measurements. In both cases, a control experiment was included lacking any test compounds and containing only an increasing concentration of ^13^C_5_, ^15^N-glutamic acid in the precipitation agent.

## Results and discussion

### Development and optimization of a cell-based ^13^C_5_, ^15^N-labeled glutamic acid uptake assay on the ADE-MS

**FIGURE 1:**
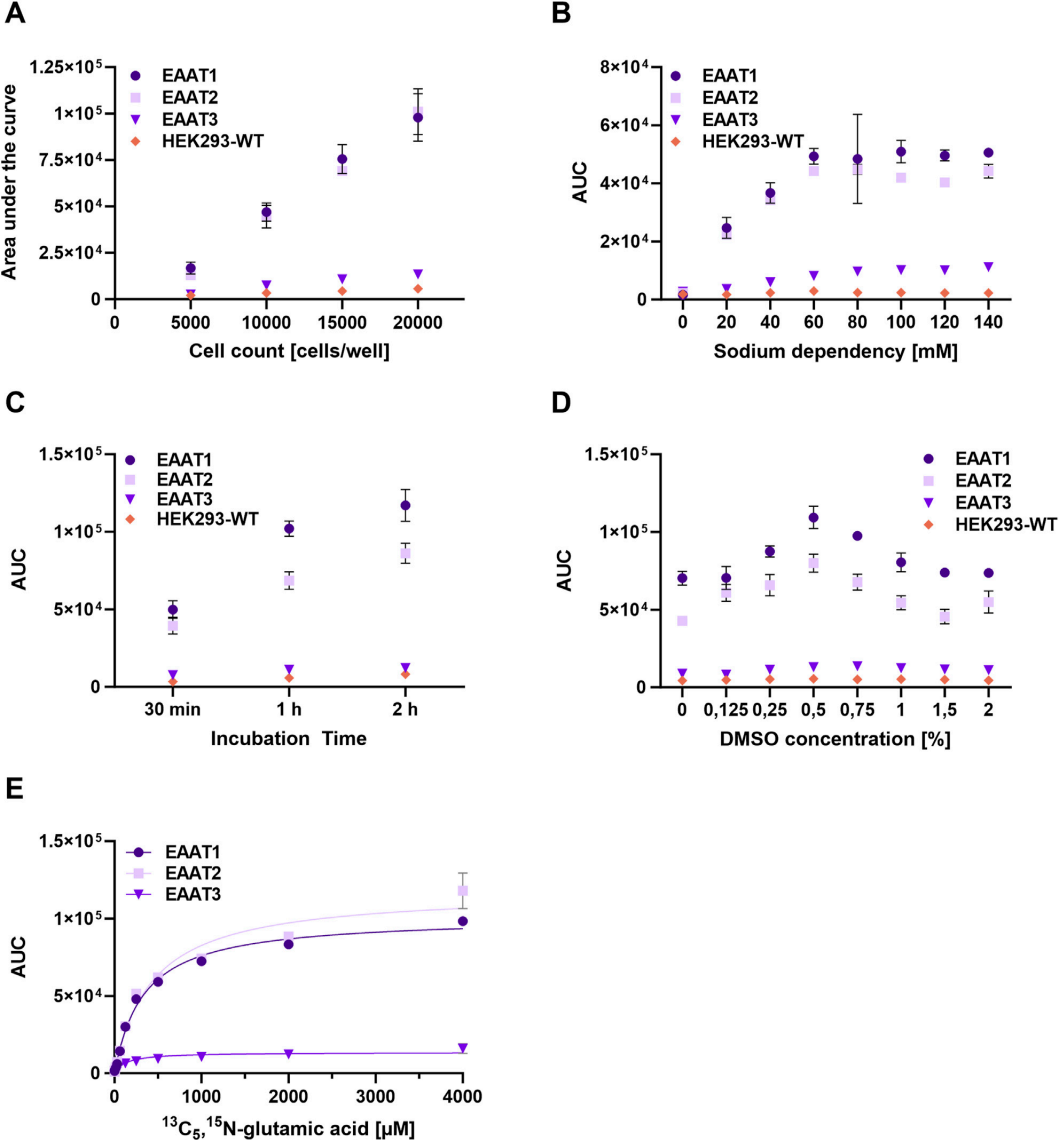
Assay Development on the ADE-MS for the transporters EAAT1-3. **(A)** Optimization of cell count. Experiments are conducted with four different cell numbers per well (5,000–20,000 cells/well). **(B)** Verifying sodium dependency and determination of optimal [Na^+^] concentration in the assay buffer. Different concentrations of sodium (0–140 mM) are tested to determine the best assay window for the transporters. **(C)** Determination of the ideal incubation time by experimenting with 30 min, 1 h, and 2 h incubation time. **(D)** Different concentrations of DMSO (ranging from 0%–2%) are tested to verify the tolerance of 1% DMSO typically used in experiments for validating and screening compounds. **(E)** Substrate dilution starting at 4 mM (10 steps, dilution factor 1:2) of labeled ^13^C_5_,^15^N- glutamic acid in activation assay buffer. K_m_ values are determined by using the Michaelis-Menten equation. EAAT1 = 308 µM (95% confidence interval: 270.3; 344.7 µM), EAAT2 = 427 µM (95% confidence interval: 345.3; 507.8 µM), EAAT3 = 224 µM (95% confidence interval: 144.1; 304.2 µM).

As a first step, experiments are conducted at four different cell concentrations per well to optimize the cell count for further experiments. Figure 1A shows the comparison of results for all three SLCs tested. Although the highest signals are detected for 20,000 cells/well, the signal-to-background (S/B) ratio indicates there is no significant difference between 15,000 and 20,000 cells/well for EAAT1 and EAAT3 (Supplementary Figure S2A). Only in the case of EAAT2 is there a wider split between both cell numbers. However, since EAAT1 demonstrates the highest signals, subsequent experiments will be performed with 15,000 cells/well for all 3 cell lines. This will facilitate handling all 3 cell lines simultaneously and allow better comparability among them.

In the second step of the assay development process, experiments are conducted at three different substrate incubation times (30 min, 1 h, and 2 h). The results, presented in Figure 1C, indicate that a 2-h incubation time yields the highest signals. However, Supplementary Figure S2C shows that both the 1-h and 2-h incubation times achieve high signals, with the 2-h duration not significantly outperforming the 1-h condition. The S/B-ratio for the 1-h incubation already demonstrates a substantial separation between the wildtype cell line and the cell lines overexpressing the SLCs (as seen in Supplementary Figure S2C). Notably, EAAT1 produces the highest signals, followed by EAAT2 at approximately 68% of EAAT1’s signal. On the other hand, EAAT3 generates a much lower signal, accounting for only about 11% of EAAT1’s signal in terms of the S/B (Supplementary Figure S2C). This pattern has been observed consistently in all the experiments conducted.

As the transport of all three SLCs is dependent on sodium, the next step is to determine the optimal sodium concentration in the assay buffer and verify its sodium dependence (Magi et al., 2019). Previous in-house studies with the transporters have shown that saturation of signal occurs at approximately 100 mM. Consequently, testing of sodium concentrations has been extended up to 140 mM, which is considered the physiological concentration of sodium in the extracellular space. This approach ensures that the sodium concentration being investigated is within the relevant range, thereby enhancing the accuracy and validity of the results obtained. The data presented in Figure 1B demonstrate a distinct sodium dependency for all three EAAT subtypes, with signal strength gradually increasing with higher sodium concentrations until reaching a plateau at 120 mM. This trend is further supported by the S/B values shown in Supplementary Figure S2B. The transporter EAAT1 (SLC1A3) displays the highest signals, even though the maximal signal is already achieved at 120 mM [Na^+^] concentration. However, to avoid any depletion of sodium that might impede glutamate transport, a concentration of 140 mM is selected for subsequent experiments for all three transporters. Notably, EAAT2 exhibits high signals at this concentration, but only about 75% of those observed for EAAT1, while EAAT3 produces only 13%.

Compounds are typically preserved by freezing and storing them in DMSO. When validating compounds, a concentration of 1% DMSO is used during the cell-based glutamic acid uptake assay. Different concentrations of DMSO (0%–2%) are added to the assay buffer to assess the DMSO tolerance of the cells. The rate of substrate import was then determined, as shown in Figure 1D. The findings indicate that a concentration of 0.75% DMSO leads to the highest signal, while the lowest signal intensities are observed for 0.125% and 2% DMSO concentrations across all three transporters. This indicates that at these DMSO concentrations, the uptake of glutamic acid seems to be altered by DMSO. Notably, 1% DMSO is well-tolerated by all 3 cell lines and leads to similar S/B levels compared to 0% DMSO (Supplementary Figure S2D). This represents a desirable outcome since it indicates that the signals are not altered by this DMSO concentration.

To determine the apparent K_m_ values of EAAT1-3 for the cell-based ^13^C_5_, ^15^N-labeled glutamic acid uptake assay, buffers containing increasing concentrations of labeled glutamic acid (ranging from 0–4 mM) were applied to the cells. The Michaelis-Menten equation is then utilized to determine the K_m_ values, with Figure 1E representing the results obtained. For EAAT1, an apparent K_m_ value of 308 µM (95% confidence interval: 270.3; 344.7 µM) is calculated for the isotope of glutamic acid. The K_m_ value for EAAT2 is calculated as 427 µM (95% confidence interval: 345.3; 507.8 µM), and for EAAT3, the value is 224 µM (95% confidence interval: 144.1; 304.2 µM). With the final assay protocol (also see Supplementary Figure S1) including critical parameters determined with 15,000 seeded cells per well, 1 h incubation time, 140 mM [Na^+^], and 300 µM of ^13^C_5_, ^15^N-labeled glutamic acid in the assay buffer, the assay undergoes validation.

### Cell-based glutamic uptake assay identifies potential activators and inhibitors for excitatory amino acid transporters

The dysregulation of Excitatory Amino Acid Transporters (EAATs) has been associated with various pathological conditions (Dvorak et al., 2021; Wang et al., 2020). Consequently, modulators that can activate EAAT function or enhance their expression have potential as therapeutic agents for these conditions (van Veggel et al., 2024). However, the availability of such activators is currently limited, and their characteristics are not well-defined. In contrast, numerous well-characterized inhibitors, both competitive and non-competitive, are available to inhibit glutamate transport. Due to the robust characterization of these inhibitors compared to emerging activators, our assay validation is based on inhibitors to demonstrate the reliability and robustness of the assay. Our ultimate objective is to develop an assay that will facilitate the identification and testing of activators in subsequent stages of research.

**FIGURE 2:**
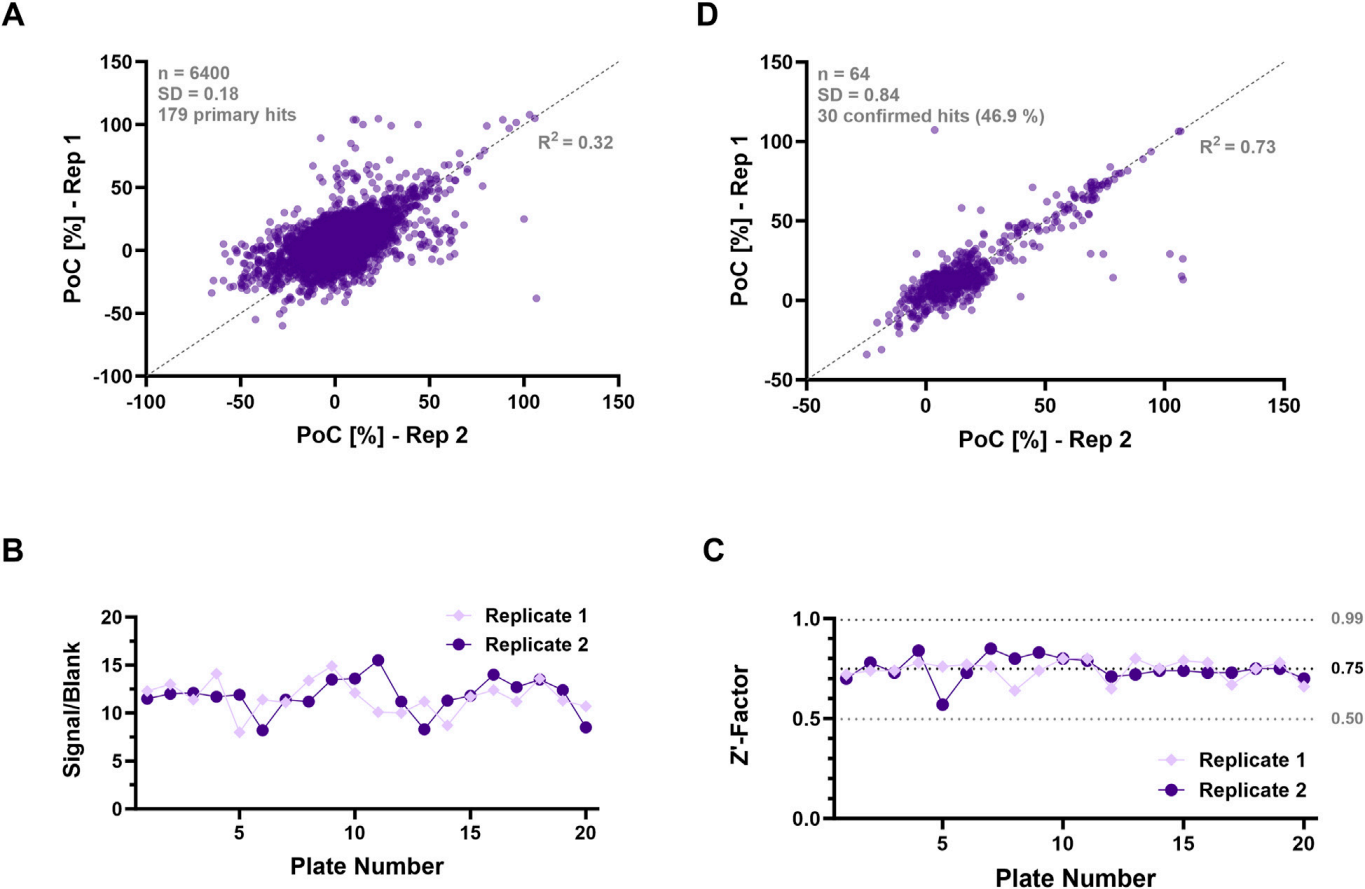
Results screening campaign for EAAT1 with ADE-MS readout and evaluation of assay quality. **(A)** Correlation plot of two individual replicates of the pilot screen (n = 6,400 compounds), also represented by *R* ^2^ = 0.32. Results of obtained inhibition values displayed in PoC [%]. Compounds undergo dose-response evaluation, starting at 30 µM (10-step dilution series, 1:2) in both replicates. Assay plates include DMSO (1%, high control) and TFB-TBOA (1 µM, inhibition control) as controls. Hit threshold of 39.5% (=3x PoC_MD_ + PoC_median_) results in 179 primary hits. Line of identity indicated in dotted line (grey). **(B)** Distribution of S/B values and **(C)** Z’ values of both replicates of the validation screening with a throughput of 20 assay plates throughout the pilot screen. The average Z’ value (black) and three-fold single deviation (light grey) are highlighted by dotted lines. **(D)** Correlation plot of the confirmation screening (n = 64 compounds), also represented by *R* ^2^ = 0.73. Compounds undergo the same treatment and conditions as in the pilot screen with a confirmation rate of 46,9%, resulting in 30 confirmed compounds. Line of identity indicated in dotted line (grey).

After successfully developing a cell-based glutamic acid uptake assay for ADE-MS readout, the assay undergoes a crucial validation step. This involves screening a subset of the Sanofi-internal validation library, comprising 6,400 compounds. Potential hits with inhibitory activity are then validated in a subsequent confirmation screening to ensure the assay’s reliability, reproducibility, and robustness. Given that EAAT1 exhibits the highest signal intensity among the three investigated SLCs and thus presents the most favorable assay window, this cell line is selected for subsequent screening experiments. The screening involves 20 compound plates with a total of 6,400 compounds tested at a concentration of 10 µM using a single-concentration approach with two replicates (40 plates for assay statistics). Figure 2A shows that both inhibitors and activating modulators were detected. Focusing on inhibitors for this purpose, hits are identified by determining the correlation between both replicates (Figure 2A). Control values are determined by calculating the average of high (DMSO 1%) and low controls (Inhibitor control TFB-TBOA, 1 µM) set at 100% and 0% activity, respectively. The response values of the wells containing compounds are normalized against these controls and expressed as a percentage of control (PoC).

The S/B values presented in the validation screen depicted in Figure 2B exhibited a greater degree of scattering. Conversely, the Z′ values shown in Figure 2C demonstrate a moderate level of variability, as they remain within a 3-fold standard deviation window. This moderate variability, characterized by a standard deviation of 0.18, underscores the robustness of the assay. The threshold for activity in the single-dose HTS screening was set at 3 × MD + median, where MD represents the mean deviation. This leads to a threshold of 39.48 PoC [%] and a hit rate of 2.8%, resulting in the identification of 179 primary hits during the screening process. The correlation was represented by a rather low value of *R* ^2^ = 0.32, indicating the scattering of the obtained PoC values (Figure 2A). However, the variability in the cell-based assay can be ascribed to the assay’s inherent nature and the cells’ robustness to withstand the assay’s conditions, such as washing and centrifugation steps. Similar fluctuations were reported by Speckmeier et al. (2022). Based on these observations, an *R* ^2^ value of 0.32 can be considered moderate in the context of the present study.

To further elucidate the observed signal variability which resulted in a rather poor replicate correlation in the primary screening (Figure 2A) and to evaluate the impact of potential matrix effects, a series of controlled experiments were conducted involving the introduction of both confirmation compounds and varying concentrations of the substrate, specifically ^13^C_5_, ^15^N glutamic acid, into the precipitation agent. These experiments utilized either lysed EAAT1 cells in the precipitation agent, consistent with the assay methodology, or the precipitation agent alone, devoid of lysed cells. Remarkably, the analysis revealed no significant effects of the compounds on the results of both approaches (see Supplementary Figures S5A–H). Additionally, a control experiment incorporating only the substrate, absent of any test compounds, demonstrated no alterations in the signal—whether utilizing lysed EAAT1 cells in the precipitation agent or the precipitation agent alone (Supplementary Figure S5M). In addition to the confirmed hit compounds, we additionally evaluated several well-characterized (tool) compounds, namely, TFB-TBOA, UCPH-101, UCPH-102, and Loratadine, for which dose-response curves and IC_50_ values have been previously published by Digles et al. (2024). These findings are also presented in Supplementary Figure S4. Importantly, no matrix effects were observed during the experiments with these compounds, as indicated in Supplementary Figures S5I–L. This evidence reinforces the conclusion that matrix effects did not significantly influence the experimental outcomes and may be considered negligible with respect to fluctuations observed during validation and compound screening.

For the confirmation screening, 64 out of 179 primary hits that demonstrated a PoC above the threshold of 39.48% in both replicates during the validation screening were selected. The results are displayed in Figure 2D The value of *R* ^2^ = 0.73 demonstrates a stronger correlation between both replicates as compared to the validation screening, establishing the reliability and reproducibility of the assay and suggesting a consistency in the ability of the replicates to confirm hits, highlighting the robustness of the screening methodology. Overall, the screening results in 30 confirmed hits out of 64 primary hits and a confirmation rate of 46.9%. While additional optimization, such as the automation of the process, could enhance consistency and minimize scattering, the assay window and Z′ values exceeding 0.5 achieved in the glutamic acid uptake assay confirm its suitability as a HTS methodology.

### Identification of potent inhibitors for transporter EAAT1 through dose-response curves analysis

Following the confirmation screening, dose-response curves are generated by evaluating the inhibitory potential of the compounds on the transporter EAAT1 at various concentrations during the glutamic acid uptake assay on the ADE-MS using the established assay protocol. This approach enables the determination of IC_50_ values, facilitating the characterization of the potency of the tested compounds. Additionally, it allows for comparison between screening technologies, thereby validating the identified hits.

**FIGURE 3:**
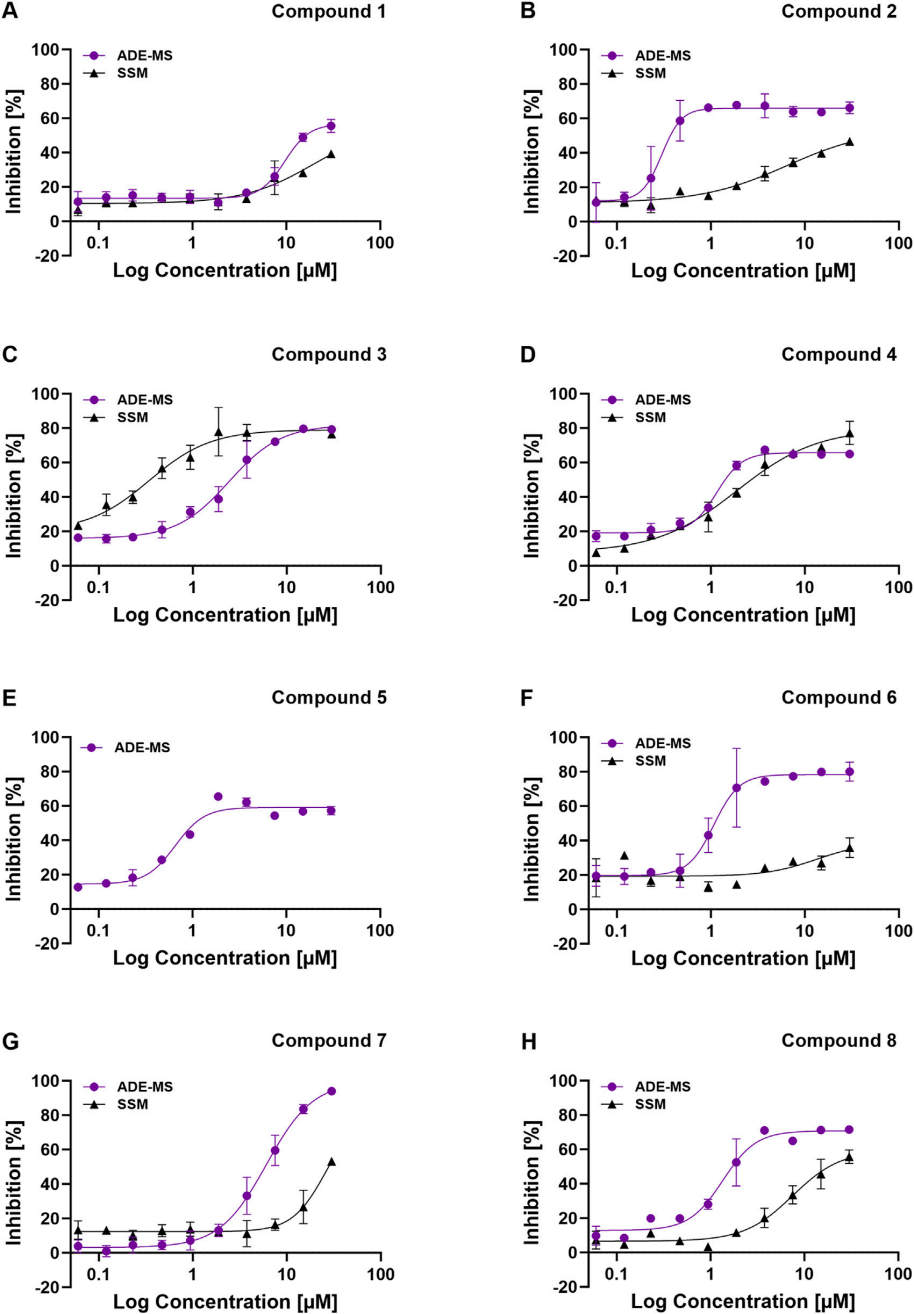
Comparative Analysis of Dose-Response Curves from ADE-MS and SSM-Based Assay Platforms. The inhibitory activity of compounds on the EAAT1 transporter is displayed in percentage [%]. Confirmation Compounds were tested in a dose-response approach with a starting concentration of 30 µM (10 steps, dilution factor 1:2). The resulting data was analyzed using GraphPad Prism to generate dose-response curves for the respective compounds, comparing both platform technologies: **(A)** Compound 1. **(B)** Compound 2. **(C)** Compound 3. **(D)** Compound 4. **(E)** Compound 5. For this compound, no dose-response curve could be generated on the SSM-based platform. **(F)** Compound 6. **(G)** Compound 7. **(H)** Compound 8. Error bars represent the standard deviation (SD) derived from two independent replicates. ADE-MS approach in purple and SSM-based platform in dark grey. Concentrations of the compounds are expressed in micromolar units (µM).

Eight confirmed hit compounds have been selected based on potent but varying IC_50_ values, representing the variability expected in a standard drug screening campaign. This approach aims to imitate the same range of possible outcomes and ensure that the selected compounds are thoroughly assessed. The dose-response curves and IC_50_ values of the respective compounds tested on the ADE-MS are summarized in Figure 3 in purple as well as in Table 1. The experimental findings reveal that Compound 2 exhibits the highest potency, characterized by a notably low IC_50_ value of 0.24 µM (Figure 3B). This is closely followed by Compound 5, which has an IC_50_ of 0.62 µM (Figure 3E). Additionally, Compounds 4 (IC_50_ = 1.13 µM), 6 (IC_50_ = 1.54 µM), and 8 (IC_50_ = 1.34 µM) demonstrate promising inhibitory activities (Figures 3D, F, H). Compound 3 shows less inhibitory activity with an IC_50_ of 2.75 µM, but remains promising (Figure 3C). In contrast, Compound 7 displays a moderate inhibitory effect with an IC_50_ of 6.06 µM, while Compound 1 shows the least inhibitory effect among all tested compounds, with an IC_50_ value of 10.78 µM (Figures 3A, G).

**TABLE 1:** Overview IC_50_ values of both screening technologies for confirmation compounds.

	SURFE^2^R		ADE-MS	
Compound	IC_50_ [µM]	CI (95%) [µM]	IC_50_ [µM]	CI (95%) [µM]
1	>30	-	10.78	(7.941; 14.63)
2	>30	-	0.239	(0.184; 0.311)
3	0.35	(0.1; 0.61)	2.752	(2.160; 3.506)
4	1.84	(1.05; 2.63)	1.128	(0.099; 1.286)
5	n.a.	n.a.	0.624	(0.481; 0.809)
6	>30	-	1.544	(1.251; 1.905)
7	>30	-	6.066	(4.921; 7.478)
8	22.5	(15.85; 29.13)	1.340	(1.059; 1.696)

Confidence intervals are calculated for SURFE^2^R data using XLfit and obtained for ADE-MS readouts by Genedata Screener analysis. CI (95%) = (lower CI (95%); upper CI (95%).

Furthermore, data obtained from the LDH Cytotoxicity Test (Supplementary Table ST1) indicate that over half of the compounds exhibit varying levels of cytotoxicity. Specifically, Compounds 1 through 4 demonstrate modest to high cytotoxic effects when assessed at the maximum concentration of 30 µM. Notably, Compounds 2 and 4 show significantly reduced cytotoxicity at a lower concentration of 10 µM. Conversely, Compounds 1 and 3 maintain cytotoxic characteristics even at lower concentrations. Therefore, the inhibition values observed in the dose-response experiments for these compounds may be confounded by their cytotoxic nature rather than their specific inhibitory activity. Compound 6 appears to exhibit low to modest cytotoxicity when tested at both 30 µM and 10 μM, which may influence its inhibitory activity. On the other hand, Compounds 5, 7, and 8 exhibit very low cytotoxicity at both the 30 μM and 10 µM concentrations. In these instances, the inhibition values derived from the experiments can be attributed to their genuine inhibitory properties rather than cytotoxic effects.

### Optimizing assay development for electrophysiological analysis of EAATs as a tertiary assay

**FIGURE 4:**
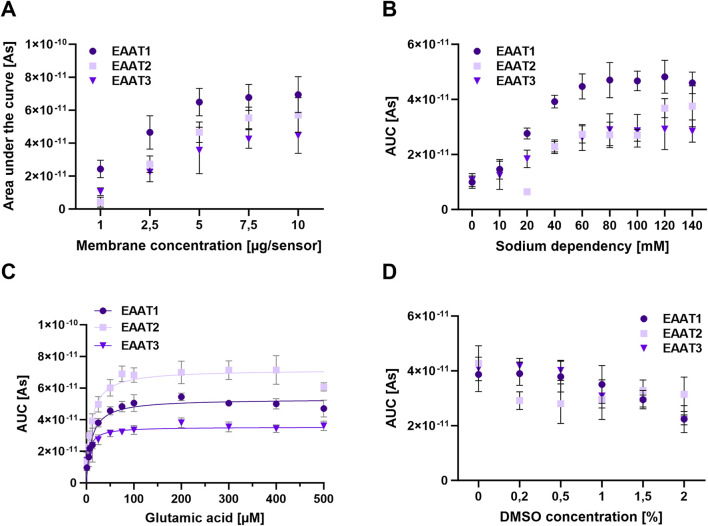
Assay Development on the SURFE^2^R 96 SE for the transporters EAAT1-3. **(A)** Determination of the optimal membrane concentration. For sensor preparation increasing concentrations of the respective membranes [1–10 μg/sensor] were applied and the induced current response was compared. **(B)** Sodium dependence. The glutamate transport activity was observed at increasing NaCl concentration in non-activating sodium buffer and activation buffer (0–140 mM). Choline was used as an inactive ion. **(C)** Glutamic acid affinity. To analyze the optimal glutamic acid affinity, the activation buffer containing different concentrations of glutamic acid (up to 500 μM) was used. Membrane concentration used for sensor preparation = 4 μg/well. The K_m_ values were determined using the Michaelis-Menten equation. EAAT1 = 10.5 μM (95% confidence interval: 6.4; 14.6), EAAT2 = 9.5 μM (95% confidence interval: 5.7; 13.4) and EAAT3 = 6.1 μM (95% confidence interval: 1.1; 11.1). **(D)** DMSO dependence. The transport activity of glutamic acid was analyzed at increasing DMSO concentrations in potassium buffer, non-activating sodium buffer and activation buffer (0%–2%). For the evaluation, the integral of the current curve was analyzed (AUC) and plotted against the concentration [μM]. The error bars represent the standard deviation.

Initially, we fine-tuned the membrane concentration used for sensor preparation. This step significantly impacts the assay’s sensitivity and is vital for ensuring reliable electrophysiological analyses when constructing the SSM sensor. Inadequate membrane preparation may lead to the formation of a perforated layer, while excessive concentration may result in a thick membrane, thereby reducing signal strength or generating artifacts (Bazzone et al., 2017). Thus, striking a balance between these two factors is crucial to achieve high-quality results. Figure 4A illustrates the results obtained from different membrane protein concentrations of HEK293 cells overexpressing EAAT1, EAAT2, or EAAT3. The data demonstrate that increasing the membrane protein concentration does not necessarily correspond to a higher signal, highlighting the importance of identifying the ideal membrane concentration for sensor preparation. For instance, for cells overexpressing EAAT1, the signal plateaus after the protein concentration reaches 5 µg/well. Similarly, a plateau in signal can be observed in cells overexpressing EAAT2 or EAAT3 at a protein concentration of 7.5 µg/well. Consequently, subsequent experiments of assay development are conducted using a concentration of 6 µg/well for all EAATs.

In the subsequent phase of assay development, the optimal substrate concentration (glutamic acid) is determined for the respective EAATs. This process involves the calculation of K_m_ using Michaelis-Menten kinetics, which is crucial for subsequent validation of compounds. Competitive inhibitors compete with the substrate for the active center on the transporter. Therefore, excessive substrate concentration may compromise the validation of the inhibitor’s effect. The K_m_ values of EAAT1, EAAT2, and EAAT3 for glutamic acid-mediated currents are determined by introducing activation buffer (A) with varying concentrations of L-glutamic acid (ranging from 0 to 500 µM) to the sensor (Figure 4C). The application of the Michaelis-Menten equation results in K_m_ values for glutamic acid of 10.5 µM for EAAT1, 9.5 µM for EAAT2, and 6.1 µM for EAAT3.

Next, we determined the optimal sodium concentration for glutamic acid transport by the EAATs. To achieve this, varying sodium concentrations (with choline being utilized as an inactive ion) are employed in both the non-activating sodium buffer and the activation buffer. Previous studies by O’Kane et al. (1999) demonstrated sodium dependence for EAAT1, EAAT2, and EAAT3. Additionally, Fumagalli et al. (2008) produced HEK293 cell lines expressing GLAST-1, and through experiments using sodium-rich and sodium-free buffers, established a clear sodium dependency, with glutamate transport declining by 90% in sodium-free buffers. Our findings indicate the presence of sodium dependence for all three EAATs (Figure 4B), with EAAT2 presenting an apparent absence of transport activity in the absence of sodium. Significantly reduced current measurements are observed in cells overexpressing EAAT1 or EAAT3 lacking NaCl (0 mM) or at low concentrations (10–20 mM) in the buffers. Optimal sodium concentrations for cells overexpressing EAAT1 or EAAT3 are determined to be between 60 and 140 mM, while for cells overexpressing EAAT2, the optimal range is found to be between 120 and 140 mM in the buffer. Subsequently, for all EAATs, a buffer with an optimized sodium concentration of 140 mM is employed for the ensuing experiments.

In the final stages of assay development, the impact of DMSO concentration on the transport of glutamic acid by EAAT1, EAAT2, and EAAT3 is analyzed. Varying DMSO concentrations ranging from 0% to 2% within the potassium buffer (R), the non-activating sodium buffer (NA), and the activation buffer (A) are used for this investigation. It is crucial to ensure uniform DMSO concentrations across all assay solutions to mitigate potential artifacts during measurement. Additionally, the efficacy of compounds in inhibiting the transporter is assessed as a post-assay development. These compounds are dissolved in 100% DMSO, with higher DMSO concentrations correlating with increased compound solubility. As a result, it is essential to identify the highest DMSO concentration within the buffer that minimizes signal loss. Drawing from the findings established with 1% DMSO, subsequent experiments of the dose-dependent activity of compounds across all three transporters are based on this concentration (Figure 4D), which represents the threshold at which signal loss is most effectively minimized.

### Characterization and validation of compounds using SSM-based electrophysiology as a tertiary assay

To ensure consistent and comparable screening, SLC1A3 (EAAT1) cell line membrane preparations were used to verify confirmation compounds from the ADE-MS method. This transporter demonstrated the best assay window among the three transporters on both the ADE-MS and SURFE^2^R platforms. As the SSM-based assay on SURFE^2^R was already established, no further parameter optimization was required.

The optimal concentration of membrane fraction was reassessed before subsequent experiments, as transporter density in the membrane can vary between batches, potentially affecting its capacity and conductivity. Following reassessment, a concentration of 8 mg/mL was determined for compound screening experiments, as shown in Supplementary Figure S3A. Given the new membrane fraction concentration, the apparent K_m_ value was reassessed, resulting in a calculated K_m_ value of 10.2 µM (95% Confidence Interval = 8.24–12.13 µM), which was subsequently used for further experiments (Supplementary Figure S3B).

After determining both parameters, the eight selected confirmation compounds were tested on the SURFE^2^R 96 SE platform to validate and characterize them by determining their IC_50_ values and comparing them to those obtained on the ADE-MS. The primary hits were tested in a dose-response approach with a starting concentration of 30 µM (10 steps, dilution factor 1:2). They were incubated for 15 min, and transport was triggered with 10.2 µM glutamic acid (K_m_) in the activation assay buffer. The dose-response curves of the confirmation compounds are summarized in Figure 3 (grey), illustrating a comparison between the results of both technologies. Table 1 summarizes the calculated IC_50_ values, including 95% Confidence Intervals, and compares them to IC_50_ values obtained in the cell-based labeled glutamic acid uptake assay on the ADE-MS. Notably, IC_50_ values obtained on the SURFE^2^R tended to be higher than those obtained on the ADE-MS. Moreover, a clear comparability regarding the potency ranking was not evident between the two assay platforms.

This discrepancy may arise from the inherent nature of the experimental design, as the cytotoxicity of the compounds does not necessarily influence the outcomes in the SURFE^2^R assay, which operates in a cell-free environment. Overall, Compound 3 (IC_50_ = 0.35 µM) exhibited the highest inhibitory activity on the SURFE^2^R, followed by Compound 4 (IC_50_ = 1.84 µM). The most potent compounds on the ADE-MS were Compound 2 (IC_50_ = 0.24 µM) and Compound 5 (IC_50_ = 0.62 µM), as shown in Table 1. Only Compounds 3 and 4 out of the eight selected confirmed hits could be validated with the SURFE^2^R, as their IC_50_ values remained sufficiently close to those obtained with the ADE-MS platform (IC_50_ = 2.75 µM and IC_50_ = 1.13 µM, respectively) to be considered consistent. This alignment is further substantiated by the profiles of the corresponding dose-response curves for both compounds, which exhibit one of the most congruent patterns within the entire compound set, as illustrated in Figures 3C,D. Unfortunately, despite multiple repetitions, a dose-response curve for Compound 5 could not be generated for the SSM-based platform due to inconsistent experimental results and artifacts (Figure 3E). The remaining compounds (1, 2, 6, and 7) exhibited IC_50_ values significantly higher than those of the validated compounds, thereby precluding confirmation of their inhibitory effects. This inconsistency is further illustrated by the discrepancies observed in the dose-response curves, as shown in Figures 3A,B,F and G where the profiles did not align. Additionally, well-characterized tool compounds, such as TFB-TBOA, UCPH-101, UCPH-102, and Loratadine, have undergone systematic evaluation, as reported by Digles et al. (2024). The dose-response curves generated from both assay platforms are depicted in Supplementary Figure S4 and IC_50_ values are summarized in Supplementary Table ST2, demonstrating the results of these investigations and highlighting the feasibility of characterizing these tool compounds using the specified methodologies.

## Discussion

In this manuscript, we describe the development of two complementary assays for glutamic acid uptake: a cell-based labeled assay using the ADE-MS platform and a solid-supported membrane (SSM)-based assay on the SURFE^2^R 96SE. Our work encompassed the optimization of critical assay parameters for both methods, followed by the screening of 6,400 compounds using the ADE-MS and subsequent validation of selected compounds through the SSM-based assay.

Results from both platforms indicate that the signal intensities corresponding to EAAT1 and EAAT2 are over sixfold greater than those of EAAT3 (Figures 1, 4). This disparity may be partially attributed to the relatively lower expression levels of SLC1A1 compared to the other overexpressed transporters in the cell lines, as demonstrated by Western blot analysis (data not shown). However, existing literature widely posits that the primary mechanism responsible for the termination of glutamatergic transmission lies in the activity of EAAT1 and EAAT2 (Bianchi et al., 2014). These transporters possess a higher affinity for glutamate than EAAT3 and are localized in glial cell processes situated in close proximity to the synaptic cleft. In contrast, EAAT3 is predominantly located extrasynaptically (Bianchi et al., 2014).

EAAT3 may play an important role in situations where glutamate spills out from the synaptic zone or reaches abnormally high extracellular concentrations, such as under ischemic conditions. It could be pivotal in glutamatergic termination in areas like the hippocampus, where most synapses are not closely surrounded by astrocytic processes. It should be noted that EAAT3 is much less abundant than EAAT2, even in these regions (Bianchi et al., 2014). Furthermore, EAAT3 is known to also transport cysteine and may provide the majority of the influx of this amino acid into neurons, where it is used for glutathione synthesis, one of the most important intracellular antioxidants that contribute significantly to neuroprotection (Kritis et al., 2015; Magi et al., 2019). Hence, it could be hypothesized that EAAT3 might have a more essential role in cysteine uptake rather than glutamate uptake, which could explain why the signals generated by EAAT3 were lower than those observed in EAAT1 and EAAT2, as EAAT3 has a comparably lower affinity towards glutamate.

The development of these two assays has enabled the comparison of both methods through the correlation of calculated K_m_ and IC_50_ values of tool inhibitors. The findings indicate that the IC_50_ values derived from the SURFE^2^R assay are generally higher than those obtained from the ADE-MS assay. Additionally, cytotoxicity assessments have demonstrated that some observed inhibitory activities may be confounded by the cytotoxic effects of the compounds, rather than indicative of a genuine inhibitory mechanism.

During the optimization of the SSM-based electrophysiology assay using the SURFE^2^R platform, the Michaelis-Menten constants K_m_ for the transporters (EAAT1-3) were determined, yielding values of 10.5 µM for EAAT1, 9.5 µM for EAAT2, and 6.1 µM for EAAT3. Notably, the newly assessed K_m_ value for the compound screening aligns closely with the previously established value for EAAT1, reinforcing its validity given the proximity of 10.5 µM. Interestingly, this value is over 30 times lower than the value calculated for EAAT1 (K_m_ = 307.5 µM) in the uptake assay on the ADE-MS (Table 2). Other studies have reported comparable K_m_ values:

**TABLE 2:** Comparison of K_m_ values of the three transporters SLC1A3, SLC1A2 and SLC1A1 determined using different assay platforms.

	SURFE^2^R		ADE-MS	
Transporter	Km [µM]	CI (95%) [µM]	Km [µM]	CI (95%) [µM]
SLC1A3 (EAAT1)	10.2	(8.24; 12.13)	307.5	(270.3; 344.7)
SLC1A2 (EAAT2)	9.5	(5.71; 13.4)	426.6	(345.3; 507.8)
SLC1A1 (EAAT3)	6.1	(2.08; 11.07)	224.2	(144.1; 304.2)

The lower and upper confidence interval are calculated with XLfit in case of SURFE^2^R data and obtained by Genedata Screener analysis for ADE-MS readouts. CI (95%)= (lower CI (95%); upper CI (95%)).

Arriza et al. (1994) conducted a comprehensive investigation to determine the Michaelis-Menten constants (K_m_) for the transporters EAAT1-3. Their findings revealed a K_m_ of 48 ± 10 µM for EAAT1, 97 ± 4 µM for EAAT2, and 62 ± 8 µM for EAAT3 through radio-labeled uptake experiments performed in transfected COS7 cells. Notably, subsequent measurements obtained via an electrogenic uptake assay using microinjected oocytes in conjunction with the Voltage-Clamp method yielded comparable yet reduced K_m_ values: 20 ± 3 µM for EAAT1, 18 ± 3 µM for EAAT2, and 28 ± 6 µM for EAAT3. These findings underscore already the influence of cellular context on the kinetic parameters of EAATs, highlighting the necessity for careful interpretation of transporter kinetics across different experimental systems (Arriza et al., 1994). In subsequent studies, Vallejo-Illarramendi et al. (2005) reported a K_m_ value of 27.5 ± 2.3 µM for EAAT1, while Canul-Tec et al. (2017) determined a K_m_ of 21 ± 10 µM for the respective transporter, both derived from glutamic acid uptake assays. Faure et al. (2006) further characterized EAAT1 with a K_m_ of 7.9 µM, EAAT2 with 21 μM, and EAAT3 with 9.9 µM in their FLIPR membrane potential assay. Additionally, Alaux et al. (2005) found K_m_ values of 9.2 µM for EAAT1, 21 µM for EAAT2, and 25 µM for EAAT3 using similar methodologies. Overall, the K_m_ value obtained in our SSM-based assay aligns with the published K_m_ values. However, the K_m_ value obtained in the uptake assay on the ADE-MS (308 µM) is significantly higher than the values reported in previous studies. This apparent discrepancy between the electrogenic and whole-cell uptake assays was previously noted by Arriza et al. (1994), who reported that the K_m_ values of the whole-cell uptake assay tend to be higher. The observed variations in results across different studies could be attributed to using different methodologies, such as the previously mentioned Voltage-Clamp method, (radio-labeled) glutamic acid uptake assays, and FLIPR membrane potential assays. It is worth noting that the SSM-based assay, which employs a cell-free method using membrane preparations, lacks the influence of the membrane potential of the cell membrane. Consequently, this methodology does not accurately reflect the physiological environment as the electrical gradient across the cell membrane is absent, and the sole driving force for substrate transport is the substrate gradient. In contrast, the ADE -MS cell-based assay is characterized by the presence of transporters in the cell membrane that are subject to various cellular factors, such as the membrane potential of the cell, the concentration of co-substrates, cofactors, posttranslational modifications, and competing molecules. These factors may significantly influence the uptake of substrates. Furthermore, differences arise between the ADE-MS and the SSM-based assay, as the latter involves a purification step of the prepared membranes that may result in a higher density of transporters in the membrane utilized for experiments. This has been observed by Sattler (2022), who reported lower K_m_ values than Gerbeth-Kreul et al. (2021), who, in turn, did not perform the purification step in their study. This higher density of transporters in the SSM-based assay may lead to a higher affinity, represented by a lower K_m_, as more transporters can efficiently uptake substrates at lower concentrations. Nevertheless, it is noteworthy that the K_m_ of the ADE-MS remains sufficiently close to that obtained on the SURFE^2^R to provide mutual confirmation.

## Conclusion

Overall, a glutamic acid uptake assay was developed on the ADE-MS platform targeting three transporters, namely, SLC1A3, SLC1A2, and SLC1A1, to characterize them further and establish an HTS-suitable screening assay for future drug discovery campaigns. The uptake assay demonstrated robustness and reproducibility and yielded 179 potential hits of inhibitory compounds on EAAT1. Out of 64 selected potential hits, 30 compounds were confirmed on the ADE-MS, and validation testing of 8 selected confirmation compounds with the SSM-based assay confirmed two compounds on the SURFE^2^R. The testing also revealed a significant K_m_ and IC_50_ disparity between the ADE-MS and SURFE^2^R platforms, as did previously published data. This variability may be attributed to the differing assay methodologies employed, the specific cell lines utilized, and inherent fluctuations in transporter expression. Importantly, the cell-free nature of the SSM-based electrophysiology platform mitigates the potential influence of compound cytotoxicity on the resulting data. Especially the discrepancies between the SURFE^2^R and ADE-MS platforms may also be attributed to differences in transporter density. A higher transporter density in the SSM-based assay due to a purification step of membrane fractions may lead to a more efficient uptake of substrates at lower concentrations. This could be demonstrated by the lower K_m_ value obtained on the SURFE^2^R than on the ADE-MS. Additionally, the SURFE^2^R platform showed higher IC_50_ values for the inhibitor TFB-TBOA, suggesting that higher concentrations of competitive inhibitors may be required to effectively compete with the substrate for binding when transporter density is more significant. This needs further to be investigated.

The findings of this research highlight the potential of utilizing the labeled glutamic acid uptake assay on the ADE-MS in HTS in drug discovery for EAAT1 inhibitors. Since ADE-MS profoundly reduces the cost of consumables, sample preparation efforts, and assay materials usually used in MS-based high-content screening techniques like MALDI- and solid phase extraction (SPE) coupled mass-spectrometry (Agilent RapidFire), it can become a real asset for future use as a primary screening platform in drug discovery projects (Speckmeier et al., 2022). Furthermore, the SURFE^2^R platform was demonstrated to be a suitable tertiary testing platform for compound validation in future drug discovery campaigns. To accurately depict the physiological processes, it would be interesting to further characterize the three transporters overexpressed in astrocytic cell lines, particularly regarding the membrane potential. It is well-known that nerve cells exhibit a more negative membrane potential (−70 mV) than somatic cells. However, before yielding significant results, it is imperative to ensure the robustness of the cells exposed to the assay conditions in the glutamic acid uptake assay.

Following this validation, the potential for utilizing this screening approach to identify activators of Excitatory Amino Acid Transporters can be assessed. This endeavor aims to discover enhancers of these transporters, which may be beneficial in both therapeutic development and further drug discovery. While the search for activators typically arises from an observed deficiency in transporter activity, it is essential to acknowledge that the identification of tool compounds is equally important. These tool compounds can provide valuable insights and facilitate subsequent research efforts, enhancing our understanding of EAATs and their role in neurobiology.

## Data Availability

The original contributions presented in the study are included in the article/Supplementary Material, further inquiries can be directed to the corresponding author.

## Supplementary material

SUPPLEMENTARY FIGURE S1 Assay Workflow on the ADE-MS. Flow chart describing the assay workflow on the ADE-MS. The process starts with seeding the induced cells (1 mg/mL doxycycline) with 15,000 cells/ well on a poly-D-lysin-treated 384 plate using the Multidrop Dispenser. Afterward, the cells are incubated at room temperature for 15 min and centrifuged at 78 *g* for 1 min. The plate is then incubated overnight at 37°C and 7.5% CO_2_. After 24 h incubation, compounds are applied (10 µL/well) using the CyBio^®^ FeliX and incubated for 15 min before 300 µM ^13^C_5_,^15^N-glutamic acid (20 µL) are applied via CyBio^®^ FeliX and incubated for 1 h at 37°C and 7.5% CO_2_. Wells are washed afterward with 70 µL/well potassium buffer using the Multidrop Dispenser, and the supernatant is discarded via Blue^®^Washer. Afterward, the precipitation process is started by adding 90 µL/well of precipitation agent with the CyBio^®^ FeliX pipetting robot. The plates are then shaken for 30 min at 500 rpm. Afterwards, a centrifuge step at 863 x g is performed for 20 min. Finally, 40 µL/well is transferred on Echo^®^ MS qualified 384 well plates via the CyBio^®^ FeliX pipetting robot and then measured with the ADE-MS.

SUPPLEMENTARY FIGURE S2 Assay development and optimization on the ADE-MS, displayed as signal to blank using the HEK293 cell wildtype as a low control for normalization. **(A)** Optimization of cell count. Experiments are conducted with four different cell numbers per well (5,000–20,000 cells/well). **(B)** Verifying sodium dependency and determination of optimal [Na^+^] concentration in the assay buffer. Different concentrations of sodium (0–140 mM) are tested to determine the best assay window for the transporters. **(C)** Determination of the ideal incubation time by experimenting with 30 min, 1 h, and 2 h incubation time. **(D)** Different concentrations of DMSO (ranging from 0% to 2%) are tested to verify the tolerance of 1% DMSO typically used in experiments for validating and screening compounds.

SUPPLEMENTARY FIGURE S3 Assay development and optimization of the SSM-based assay conditions on the SURFE2R. **(A)** Different membrane concentrations are used for sensor preparation. Increasing amounts of total protein (1–20 mg/mL) are applied to the sensor, and the induced currents are compared. The integral of the curve (AUC) is analyzed. **(B)** Glutamic acid affinity for SLC1A3 (EAAT1) is measured by applying activation buffer containing increasing concentration of glutamic acid (up to 200 µM) for the SSM-based SURFE^2^R assay. K_m_ value is calculated by using the Michaelis-Menten equation. Error bars represent mean deviation of median values of the integral of the curves.

SUPPLEMENTARY FIGURE S4 Inhibitory activity of published compounds ADE-MS vs. SSM [%]. For the SSM-based approach, compounds are tested in a dose-response approach in a 10-step dilution series, dilution factor 1:3 with a starting concentration of 30 µM for all compounds. For the ADE-MS approach, compounds are tested in a dose-response approach 10-step dilution series, dilution factor 1:2 and also a start concentration of 30 µM. The plates included the wildtype HEK293 cell line as low and DMSO 1% as high controls. Response values of the wells containing compounds are normalized against these controls and expressed as inhibitory activity in %. Error bars represent the SD of two individual replicates. [µM] = log compound concentration in micromolar units. ADE-MS approach in purple and SSM-based electrophysiology in dark grey. Data is published in Digles et al., 2024.ev.

SUPPLEMENTARY FIGURE S5 Effect of Cell-Matrix and Compound-Matrix. Investigating matrix effects by adding increasing concentrations of ^13^C_5_, ^15^N-labeled glutamic acid (0.15–300 µM) and respective compounds (10 µM) either in precipitation agent containing lysed EAAT1 cells (shown in purple) or only in precipitation agent (orange), lacking EAAT1 cells. **(A)** Confirmation Compound 1 **(B)** Confirmation Compound 2 **(C)** Confirmation Compound 3 **(D)** Confirmation Compound 4 **(E)** Confirmation Compound 5 **(F)** Confirmation Compound 6 **(G)** Confirmation Compound 7 **(H)** Confirmation Compound 8 **(I)** TFB-TBOA **(J)** UCPH-101 **(K)** UCPH-102 **(L)** Loratadin **(M)** Without compound (control).
