# Supplementary material for: The development of a high-throughput acoustic droplet ejection mass-spectrometry assay and a solid-supported membrane (SSM)-based electrophysiological assay to study the pharmacological inhibition of SLC1-A3, -A2 and -A1 in a drug discovery program
Source: Front Pharmacol. 2025 Apr 16;16:1544682. doi: 10.3389/fphar.2025.1544682 (PMC12041765; doi:10.3389/fphar.2025.1544682)
Supplement: Supplementary file 1 [file Table1.docx]

**Supplement Table T1: Results of the LDH Cytotoxicity Assay.** First Hep-G2 cells are plated in a 384-well Assay Ready Plate (15,000cells/well) in 50 µL and then incubated without lids for 20-24h at 37 °C, 7.5% CO2 and 95% humidity. The plate containing pre-dispensed compounds (10 mM, 150 nL/well, resulting in a final concentration of 30 µM) is centrifuged at 1,100 rpm for 1 minute. From each well, 20 µL of supernatant is transferred to a new assay plate with VPrep, followed by 20 µL of freshly prepared LDH reaction mixture with the Multidrop Combi (standard cassette). After incubation for 15-20 minutes at room temperature under a black lid, absorbance is measured at 492 nm using a PheraStar FSX plate reader.

|  | **30 µM LDH Tox 1%** | | **10 µM LDH Tox 1%** | |
| --- | --- | --- | --- | --- |
| **Compounds** | **Replicate 1** | **Replicate 2** | **Replicate 1** | **Replicate 2** |
| 1 | 93,0 | 91,8 | 80,5 | 73,1 |
| 2 | 47,1 | 49,6 | 1,5 | 4,3 |
| 3 | 45,6 | 43,8 | 50,6 | 49,6 |
| 4 | 59,4 | 62,8 | 13,3 | 11,3 |
| 5 | 2,1 | -3,2 | -2,6 | -3,5 |
| 6 | 32,0 | 34,9 | 9,7 | 33,3 |
| 7 | 3,8 | 3,8 | 6,4 | -1,5 |
| 8 | 1,8 | -2,1 | 1,5 | 0,5 |

**Supplement Table T2: Overview IC_50_ values of both screening technologies for tool compounds.** Confidence intervals are calculated for SURFE^2^R data using XLfit and obtained for ADE-MS readouts by Genedata Screener analysis. CI (95%) = (lower CI (95%); upper CI (95%)

|  | **SURFE^2^R** | | **ADE-MS** | |
| --- | --- | --- | --- | --- |
| **Compound** | **IC_50_ [µM]** | **CI (95%) [µM]** | **IC_50_ [µM]** | **CI (95%) [µM]** |
| TFB-TBOA | 9.354 | (6.02; 12.71) | 0.0081 | (0.0041; 0.0110) |
| UCPH-101 | 0.353 | (0.28; 0.43) | 0.509 | (0.421; 0.615) |
| UCPH-102 | 1.546 | (0.56; 2.53) | 0.135 | (0.088; 0.209) |
| Loratadine | 21.84 | (18.51; 25.18) | 6.52 | (4.706; 9.025) |
