# Supplementary figures and images for: The development of a high-throughput acoustic droplet ejection mass-spectrometry assay and a solid-supported membrane (SSM)-based electrophysiological assay to study the pharmacological inhibition of SLC1-A3, -A2 and -A1 in a drug discovery program
Source: Front Pharmacol. 2025 Apr 16;16:1544682. doi: 10.3389/fphar.2025.1544682 (PMC12041765; doi:10.3389/fphar.2025.1544682)

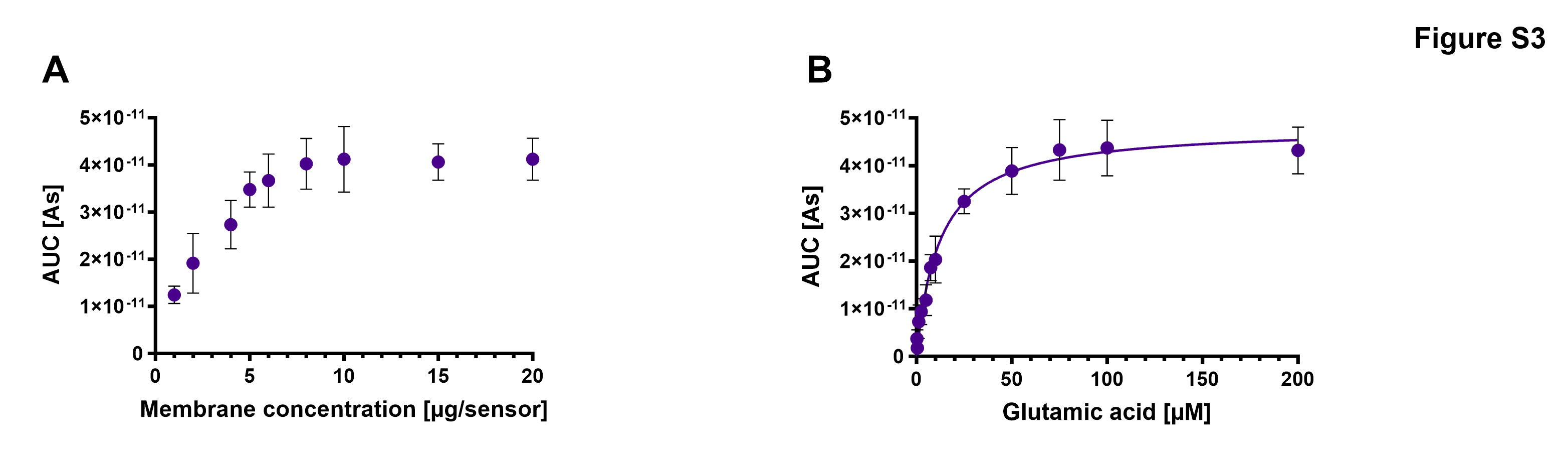

Supplement: Supplementary file 2 [file Image3.tif]

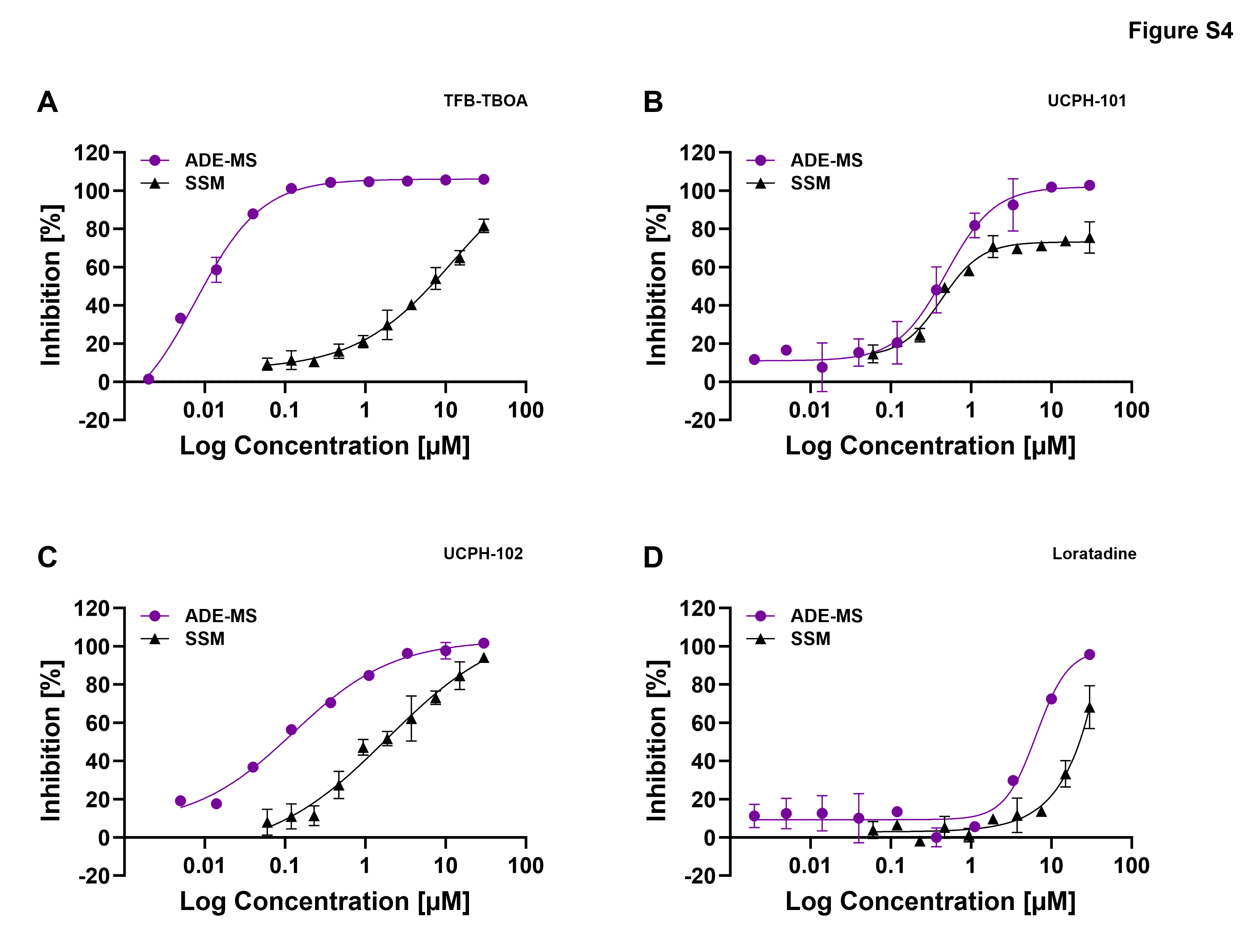

Supplement: Supplementary file 3 [file Image4.tif]

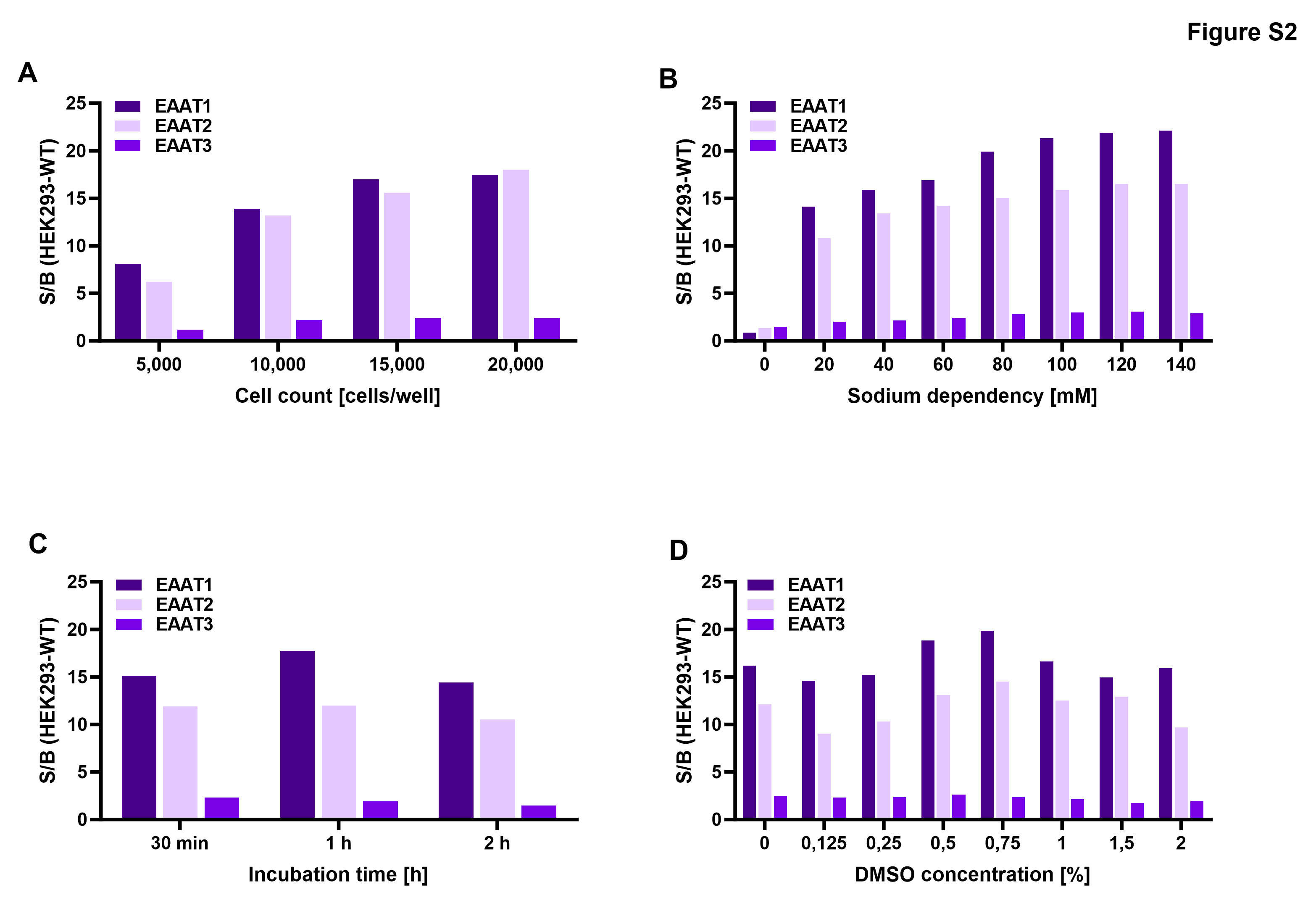

Supplement: Supplementary file 4 [file Image2.tif]

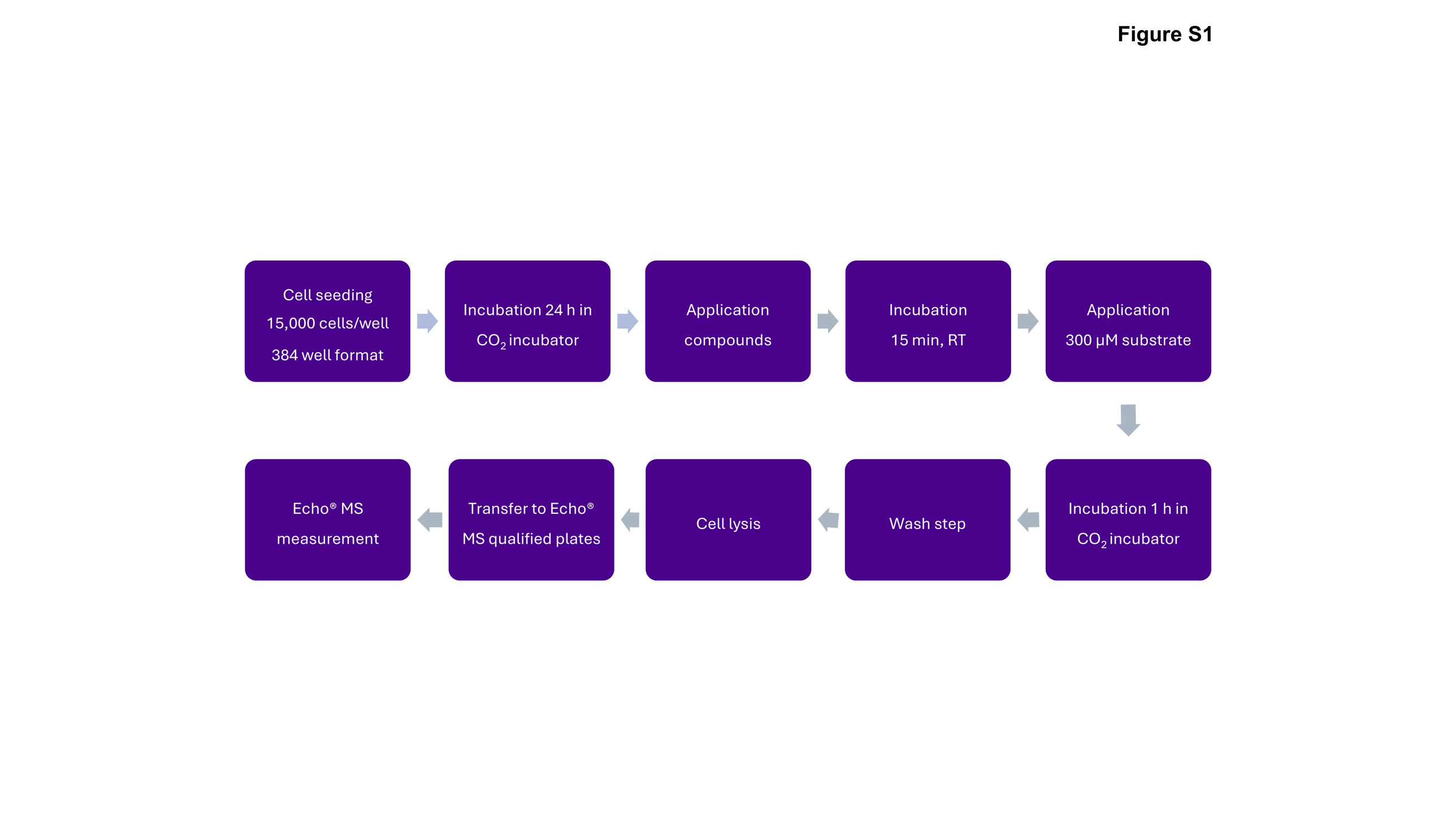

Supplement: Supplementary file 5 [file Image1.tif]

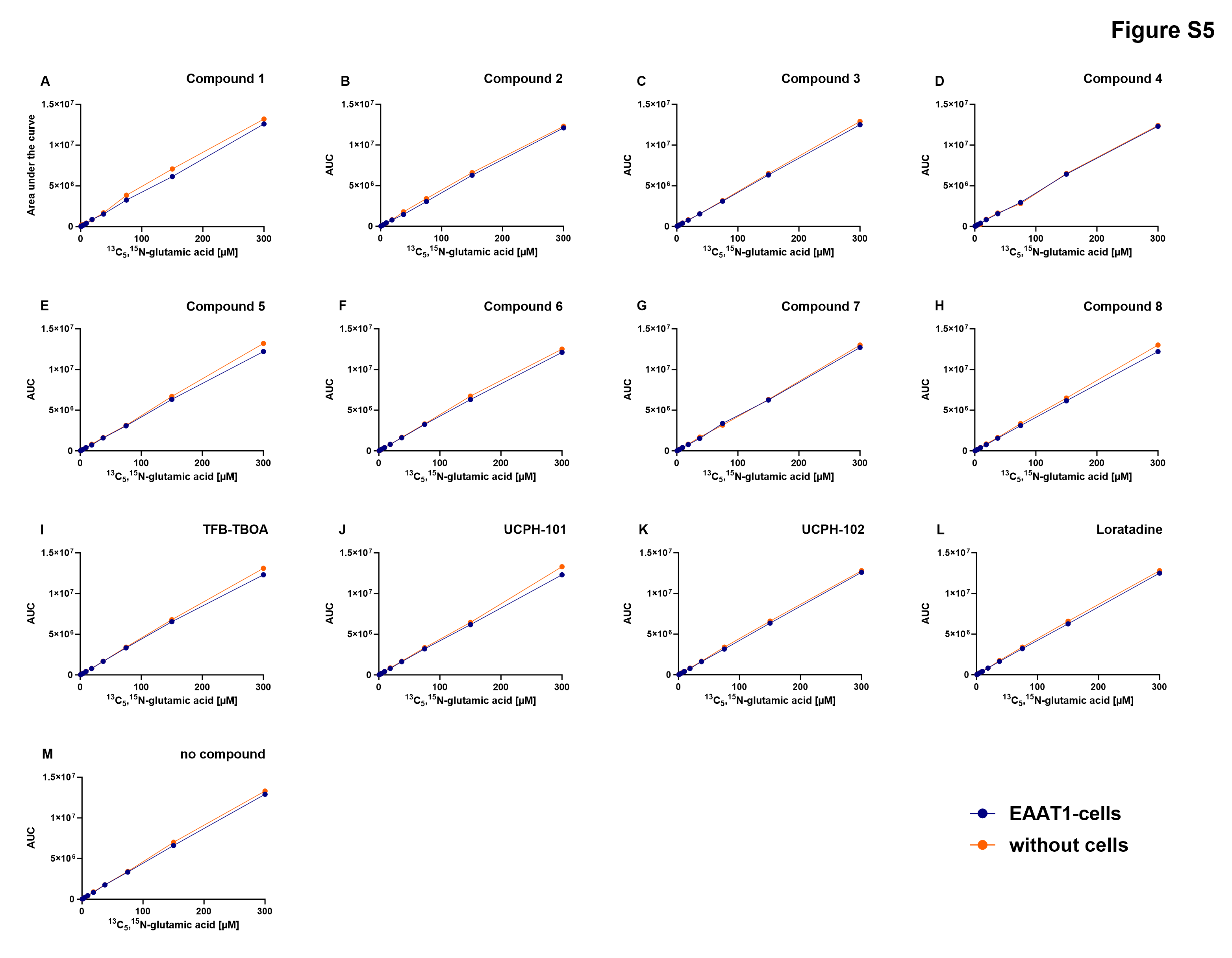

Supplement: Supplementary file 6 [file Image5.tif]
